# Robotic assisted simple prostatectomy mitigates perioperative morbidity compared to open simple prostatectomy - a single institution report

**DOI:** 10.1186/s12894-024-01615-4

**Published:** 2024-10-10

**Authors:** Magnus Larsen, Anneli Giske, Marius Roaldsen, Dag Gullan, Erling Aarsaether

**Affiliations:** 1 Department of Urology, University Hospital of North Norway, Tromsø, Norway; 2 UiT – the Arctic University of Norway, Tromsø, Norway; 3 Department of Urology, Stavanger University Hospital, Stavanger, Norway

**Keywords:** Benign prostatic hyperplasia, Lower urinary tract symptoms, Open simple prostatectomy, Robotic assisted simple prostatectomy

## Abstract

**Background:**

According to the guidelines of the European Association of Urology, open simple prostatectomy should be offered to men with a prostate size exceeding 80 mL suffering from moderate to severe LUTS in the absence of a transurethral enucleation technique. However, open simple prostatectomy is associated with complications such as bleeding, blood transfusions and increased length of stay compared to minimally invasive procedures. The aim of the study was to compare perioperative data from the first cases of robotic assisted simple prostatectomy (RASP) to that of patients subjected to open simple prostatectomy (OSP) at our department.

**Methods:**

The patients were identified by a search for the respective procedure codes. In the OSP group enucleation of the adenoma was performed through the prostatic capsule (Millin procedure), while access to the adenoma was gained through the bladder in the RASP group. Complications were scored according to the Clavien-Dindo classification system.

**Results:**

27 patients who underwent OSP were retrospectively identified and compared to the first 26 patients who were subjected to RASP. The groups were similar with respect to age, body mass index and ASA score. Operative time was significantly shorter in the OSP group compared to the RASP group. Bleeding volume, drop in postoperative hemoglobin and the number of blood transfusions were all significantly higher in the OSP group compared to the RASP group. Average length of stay was 5.5 (2–18) days in the OSP group compared to 1.6 (1–5) days in the RASP group (*p* < 0.001). The number of postoperative complications, Clavien-Dindo ≥ 2, were significantly higher in the OSP group (11) compared to the RASP group (none, *p* < 0.001).

**Conclusions:**

The introduction of robotic assisted simple prostatectomy reduced perioperative morbidity at our department.

## Introduction

Choosing the optimal surgical treatment of bladder outlet obstruction in patients with large prostate glands (> 80 mL) represents a challenge, due to the lack of randomized controlled trials available [1]. Open simple prostatectomy (OSP) is the oldest surgical treatment method and provides excellent symptom relief, improved quality of life and demonstrates durable results [2, 3]. Although long term results of OSP are favorable, minimally invasive surgical techniques have been carried forward by the desire to develop less invasive surgical methods [4, 5]. In spite of the growing evidence which suggests that RASP reduces the perioperative morbidity associated with OSP [6, 7], European guidelines still recommend open simple prostatectomy to men with a prostate size exceeding 80 mL suffering from moderate to severe lower urinary tract symptoms (LUTS), in the absence of a transurethral enucleation technique [1]. The aim of the study was to retrospectively compare perioperative data from the first 26 robotic assisted simple prostatectomy (RASP) cases to that of patients subjected to open simple prostatectomy (OSP) within the last 7 years at our department.

## Materials and methods

The study was approved by the Regional Ethical Committee at the University Hospital of North Norway. Inclusion criteria were the following: All patients who underwent either OSP or RASP between January 1st 2017 and January 1st 2024. Exclusion criteria were as follows: Patients` whose surgical procedures were erroneously coded as OSP or RASP, i.e. the description of the surgical procedure revealed that they had undergone a different surgical procedure than those presented here. The patients were identified by a search for the procedure codes in the electronic journal system (DIPS, Distribuert Informasjons- og Pasientdatasystem i Sykehus, Bodoe, Norway) and retrospectively included.

In the OSP group access to the prostate was achieved through a low midline infraumbilical incision, which was extended below the pubic symphysis. The adenoma was exposed through a transverse capsular incision and bluntly dissected with the index finger as described by Millin [8]. The bladder neck was sutured to the posterior surface of the prostatic capsule and a two-way catheter (22 or 24 French) was inserted. A Jackson-Pratt drain was left in the space of Retzius in front of the sutured prostatic capsule and brought out through a separate incision in the left or right lower abdominal quadrant.

**Fig. 1:**
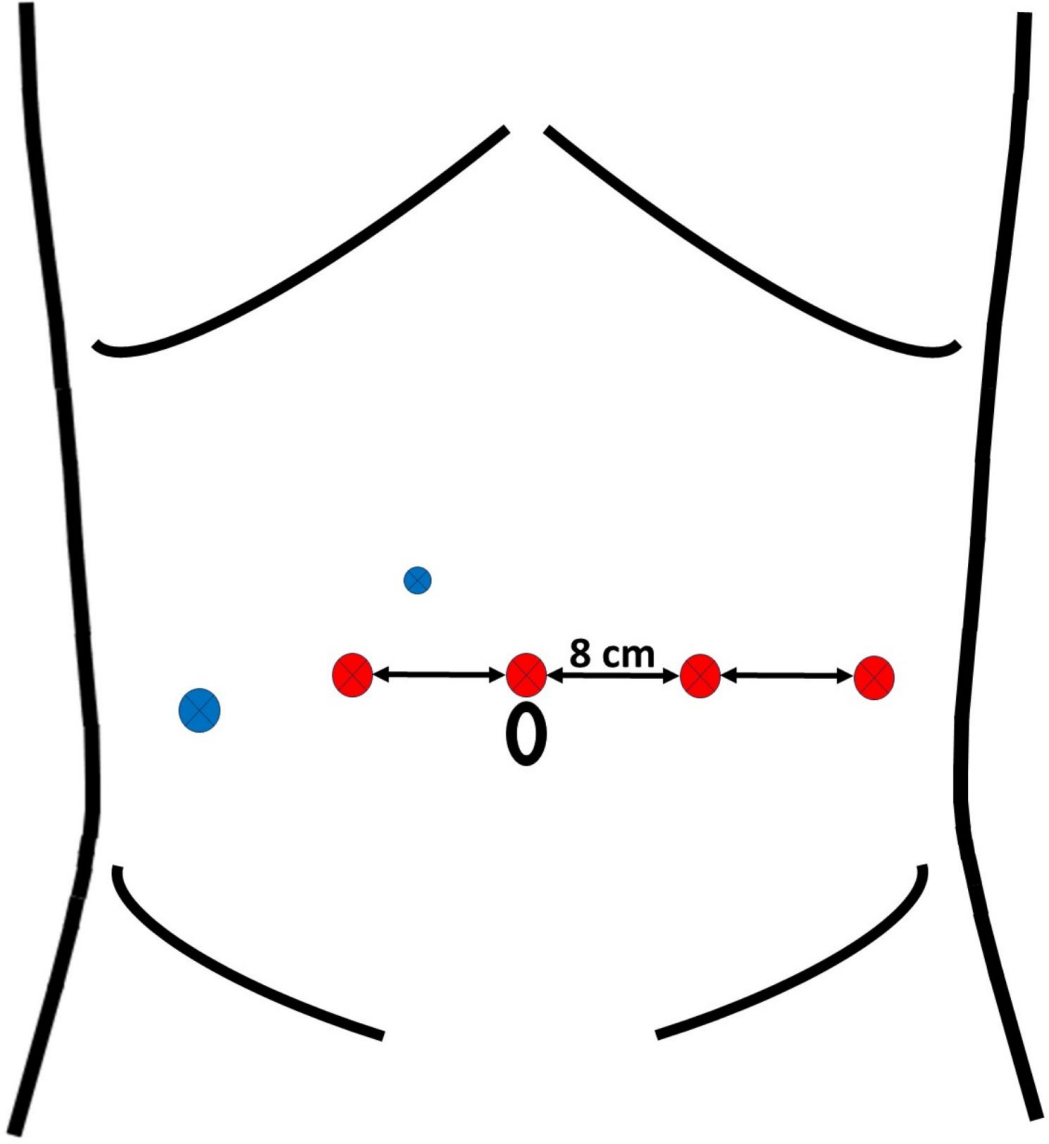
Figure 1 illustrates the port placement in RASP utilized in the present study. The robotic camera port is placed in the midline, just above the umbilicus. The additional robotic ports marked in red are inserted in a horizontal line with 8 cm of space in between. A 5 mm port (blue, small circle) is introduced between the camera port and the lateral robotic port on the patient`s right hand side, which is mainly utilized for suction. An additional 12 mm port for assistance is inserted in the right lower abdominal quadrant (blue, large circle)

**Fig. 2:**
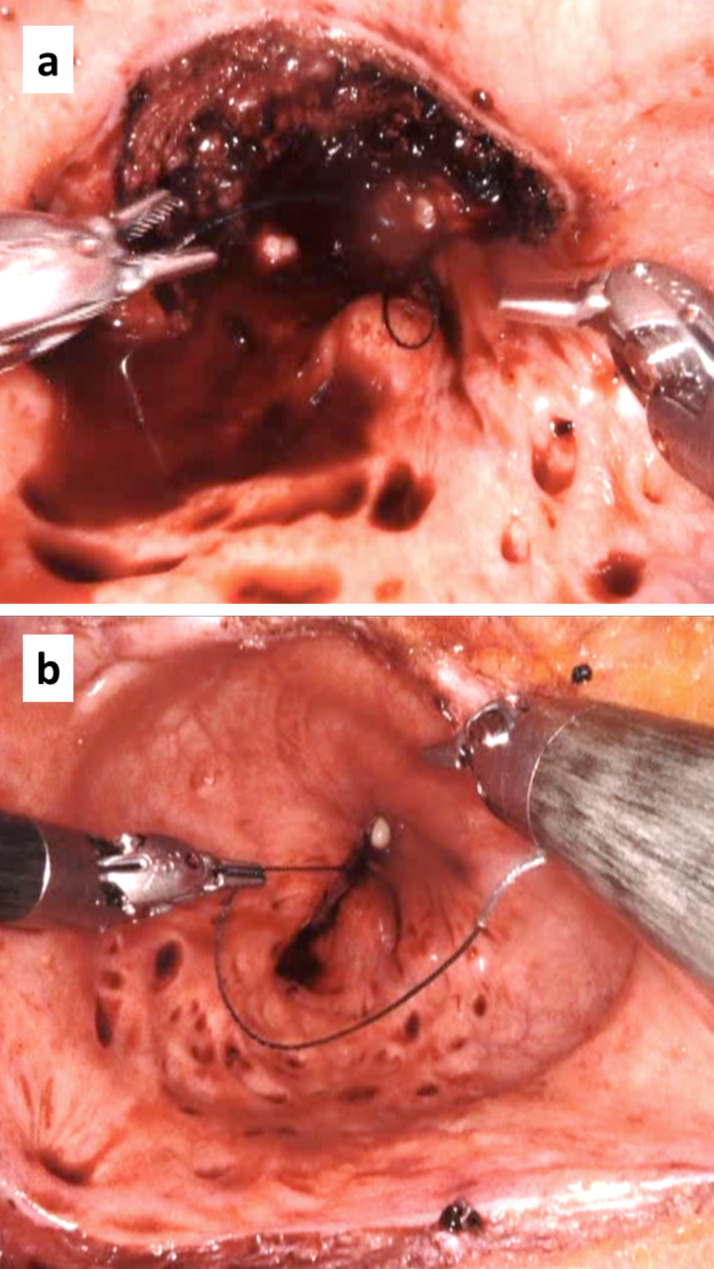
The figure illustrates how the hemostasis improves in RASP during trigonization of the bladder after the prostatic adenoma is removed. In the top (**a**) bleeding obscures the operating field. However, when the bladder neck is approximated to the urethra with a unidirectional barbed suture, bleeding control improves substantially (**b**)

In the RASP group all procedures were performed with the da Vinci XI^®^ or da Vinci X systems (Intuitive Surgical Inc., Sunnyvale, CA, USA), using a standardized 4-arm transperitoneal 6-port approach with a 0° lens, a maryland bipolar forceps, monopolar curved scissors and a ProGrasp™ forceps (Intuitive Surgical Inc., Sunnyvale, CA, USA). The port placement is depicted in Fig. 1. In this group the prostate was approached through a vertical incision in the bladder and the adenoma was dissected from the prostatic capsule with the robotic scissors, utilizing a combination of monopolar coagulation and blunt dissection. After the successful removal of the adenoma, trigonization of the bladder was achieved (Fig. 2) by suturing the edges of the bladder neck to the urethra with a unidirectional barbed suture (VLOC, 3 − 0, Covidien, Dublin, Ireland). The anastomosis was stitched from either three o`clock to nine o`clock, i.e. 180 degrees, or as a full circular 360 degree anastomosis according to each individual surgeons` preference. An 18 French two-way catheter was inserted into the bladder. Drains were avoided in all patients.

The 30 day complication rate was retrospectively collected and graded according to the Clavien-Dindo classification system [9]. Statistical analysis was performed with the IBM SPSS software (Chicago, Ill). Comparisons of numerical variables between the groups were calculated with the t test. Between-group comparisons for categorical variables were analyzed with the Pearson chi square test or the exact test. Data are presented as mean, range and standard deviation. *P* < 0.05 was considered statistically significant.

## Results

We retrospectively identified 27 patients who underwent OSP and subsequently compared these cases to the first 26 who were subjected to RASP. None of the patients had previously undergone surgical treatment for benign prostate hyperplasia. The groups were similar with respect to age, body mass index and ASA score (Table 1). There were significantly more patients in the OSP group (15 from 27) who presented with urinary retention preoperatively compared to the RASP group (6 from 26, *p* < 0.05). No significant differences between the groups were found with respect to preoperative estimated prostate volume or maximum flow rate. The preoperatively assessed international prostate symptom score (IPSS) was significantly higher in the RASP group compared to the OSP group (*p* < 0.05). Between group differences were also found with respect to preoperative prostate specific antigen (PSA). PSA was significantly higher in the OSP group compared to the RASP group before surgery, probably as a consequence of more patients presenting with urinary retention in this group.

**Table 1:** Preoperative data

	OSP (*n* = 27)	RASP (*n* = 26)	*p*
Age (years)	72 ± 6	72 ± 6	0.9
ASA score	2.3 ± 0.6	2.3 ± 0.5	0.8
BMI	27.4 ± 4	27.1 ± 3	1.0
Preoperative urinary retention	15	6	< 0.05
Preoperative estimated prostate volume (mL)	153 ± 59	130 ± 56	0.2
Preoperative IPSS	21.1 ± 6.8	29.3 ± 6.5	< 0.05
Maximum flow rate (mL/s)	8.6 ± 5.4	8.2 ± 2.8	0.8
Preoperative PSA (µg/L)	10.6 ± 7.9	5.5 ± 4.2	< 0.01

ASA = American society of anaesthesiologists, BMI = Body mass index, IPSS = International prostate symptom score, OSP = Open simple prostatectomy, PSA = Prostate specific antigen, RASP = Robotic assisted simple prostatectomy

Perioperative data are provided in Table 2. Operative time was significantly shorter in the OSP group compared to the RASP group (*p* < 0.01). Mean bleeding volume was 585 ± 317 ml in the OSP group compared to 134 ± 73 ml in the RASP group (*p* < 0.001). There were no differences between the groups with respect to preoperative hemoglobin levels. However, the drop in hemoglobin following surgery was more pronounced in the OSP group (3.5 ± 1.7) compared to the RASP group (1.7 ± 1.1, *p* < 0.001). The weight of the prostatic adenoma specimen was 129 g (range 53–372) in the OSP group compared to 86 g (20–201) in the RASP group (*p* < 0.05). The number of blood transfusions were significantly higher in the OSP group (12) compared to the RASP group (none, *p* < 0.05). The number of patients with a Clavien-Dindo score of 2 or more within 30 days were also higher in the OSP group (11) compared to the RASP group (none, *p* < 0.001). Patients in the OSP group exhibited longer hospital stays than those in the RASP group, with a mean hospital stay of 5.5 days (range 2–18) postoperatively compared to 1.6 days (range 1–5) in the RASP group (*p* < 0.001).

**Table 2:** Perioperative data

	OSP (*n* = 27)	RASP (*n* = 26)	*p*
Time of surgery (min)	100 ± 24	116 ± 29	< 0.01
Bleeding (ml)	585 ± 317	134 ± 73	< 0.001
Preoperative hemoglobin (g/dL)	14.2 ± 1.4	14.4 ± 1.1	0.6
Postoperative hemoglobin (g/dL)	10.7 ± 1.8	12.8 ± 1.3	< 0.001
Drop in hemoglobin (g/dL)	3.5 ± 1.7	1.7 ± 1.1	< 0.001
Weight of resected prostatic adenoma (g)	129 (53–372)	86 (60–201)	< 0.05
Blod transfusions (no.)	12	0	< 0.05
Length of stay (days)	5.5 (2–18)	1.6 (1–5)	< 0.001
Clavien-Dindo 2 or higher	11	0	< 0.001
Postoperative urinary retention	6	1	0.1

OSP = Open simple prostatectomy, RASP = Robotic assisted simple prostatectomy

## Discussion

These data suggest that RASP may lower perioperative morbidity compared to that of OSP with shorter length of stay, less blood loss and fewer complications as a result of changing the surgical approach from open to robotic. However, due to the relatively low number of cases, these data must be interpreted cautiously. OSP is the oldest surgical treatment of moderate to severe LUTS due to benign prostatic hyperplasia and provides an effective reduction of LUTS, increases maximum urinary flow and improves quality of life [1–3, 10–12].

However, OSP is associated with bleeding complications with estimated transfusion rates of 7–14% of reported case series [2, 10, 13, 14]. Previous studies comparing OSP with RASP has similarly to our results demonstrated a benefit of less bleeding as an advantage of the RASP procedure [5, 15–18]. In our experience, the hemostasis during enucleation of the prostatic adenoma is often hard to maintain in both these surgical procedures, in which multiple vessels between the adenoma and the prostatic capsule are divided. In contrast to OSP, however, improved visibility provided by the da Vinci Surgical system facilitates the trigonization of the bladder, which subsequently leads to adequate hemostasis as illustrated in Fig. 1. The lower bleeding volumes in RASP is also the reason why 18 French two-way catheters are routinely utilized instead of catheters with 22–24 French diameters, which are recommended in OSP. Similarly, RASP patients are transferred from the operating theatre without a drain in the space of Retzius, while this is considered mandatory to patients who have been subjected to OSP.

Opponents of the RASP procedure have previously pointed out that the surgery is more time consuming and more costly than that of OSP [15]. Our data also demonstrate, as has been argued, that operating time was significantly longer in the RASP group compared to the OSP group. However, we underline that operating times reported in this study include the learning curve for the RASP procedure. When we compared the operating time of the first 13 RASP cases (130 ± 28 min) to the last 13 (103 ± 23 min), we found that the operating time actually dropped by an average of 27 min, which is almost identical to the operating time in the OSP group (100 ± 24 min). As has been demonstrated for other robotic procedures, operating time for RASP is likely to decrease until the learning curve of the robotic procedure reaches a plateau phase. The learning curve for RASP has previously been suggested to be 10–12 cases for experienced robotic surgeons [19].

As shown in Table 2, the weight of the resected adenoma was significantly lower in the RASP group compared to what was removed in the OSP group. This would seem to suggest that RASP is a less effective method than OSP. However, when the weight of the resected adenoma was adjusted for differences in preoperative estimated prostatic volume, the differences in weight of the resected prostatic adenoma between the groups were no longer statistically significant. We speculate that the threshold for referring patients with relatively smaller prostatic volumes to simple prostatectomy has been lower in the RASP group, precisely due to the significant drop in perioperative morbidity which have been demonstrated in this group. For the same reasons, we believe that patients with relatively small prostates in the OSP era may have been more likely referred to transurethral resection of the prostate, due to concerns about perioperative morbidity, leading to a higher although non-significant, mean estimated preoperative prostate volume in the OSP group.

The cost of OSP versus the cost of RASP has been a subject of debate, with conflicting conclusions, depending on whether the length of stay and or complications are included in the calculations [15]. The present study indicates that an average of 4 days of hospitalization can be deducted from the accounting in the RASP group, which in Norwegian health care equals approximately 8000 USD. If the cost of the complications in the present study are added to the estimated cost analysis in the OSP group, we believe that RASP comes out as the favorable alternative, although a detailed cost analysis of the respective procedures was beyond the scope of this work.

Limitations of the study include the low number of cases in each of the study groups and the retrospective design. The data are collected from a single institution, and should therefore be interpreted cautiously. However, the differences in perioperative morbidity between the groups in this study are quite overwhelmingly in favour of RASP compared to OSP, in spite of the fact that the learning curve of RASP is included herein. We argue that RASP should be preferred to OSP in the absence of an effective transurethral enucleation technique and suggest that the increased costs of robotic surgery should be weighed against the benefit associated with a significant drop in perioperative morbidity.

## Data Availability

The data that support the findings of this study are not openly available due to reasons of sensitivity and are available from the corresponding author upon reasonable request. Data are located in controlled access data storage at the University Hospital of North Norway.
